# Risk factors for postoperative delirium in patients undergoing orthopedic procedures: a systematic review and meta-analysis

**DOI:** 10.1371/journal.pone.0321025

**Published:** 2025-04-01

**Authors:** Rio Suzuki, Aina Nakanishi, Masahiro Masuya, Keiko Fukuroku, Yukari Taneda, Yutaka Matsuura

**Affiliations:** 1 Mie University Hospital, Tsu, Mie, Japan; 2 Kariya Toyota general hospital, Kariya, Aichi, Japan; 3 Division of Nursing, Mie University Graduate School of Medicine, Tsu, Mie, Japan; Tehran University of Medical Sciences Endocrinology and Metabolism Research Institute, IRAN, ISLAMIC REPUBLIC OF

## Abstract

Delirium is a common complication in surgical patients following operative procedures; it often occurs in patients undergoing lower-extremity surgery. It is essential to identify and prevent the risk factors for postoperative delirium (POD) in these cases. We aimed to determine the risk factors for POD in patients who underwent lower-extremity surgery through a systematic review and meta-analysis. We included observational studies identifying risk factors for POD in patients undergoing orthopedic surgery. Data sources included the Cumulative Index to Nursing and Allied Health Literature and MEDLINE. We extracted the variables related to delirium that were analyzed by two or more studies meeting the eligibility criteria. A random-effects model was used to calculate the pooled odds ratio, standardized mean difference, and 95% confidence interval. Data were considered significant when p <  0.05. Twenty-seven studies with a total sample size of 9,044 were evaluated. Our meta-analysis revealed 20 risk factors for patients with POD undergoing orthopedic surgery, including age, cognitive scores, various preoperative laboratory values (such as serum albumin, C-reactive protein, and thyroid hormones), length of hospital stay, surgery and anesthesia duration, blood transfusion, and previous health conditions such as dementia and cardiovascular disease. Gathering preoperative and postoperative data was crucial for identifying high-risk patients for POD. In addition, preventive measures targeting POD risk factors could reduce its occurrence after orthopedic surgery.

## Introduction

Delirium, characterized by disturbances in attention, acute changes from baseline attention and awareness, and disturbances in cognition, is a common complication in patients who have undergone surgery [1]. Incidence rates vary across clinical departments, with orthopedic surgery patients experiencing delirium ranging from 12% to 51%, notably higher than the 11% and 14% observed in general ward adimission[2]. Delirium is particularly common in patients undergoing lower-extremity surgery. For instance, the incidence rates among patients undergoing representative surgeries such as total hip arthroplasty (THA) and total knee arthroplasty (TKA) range from 4% to 53% [3] and 48.1% [4], respectively. Additionally, postoperative delirium (POD) stands out as the most frequent complication in patients with femoral neck fractures [5]. According to the Japanese Orthopedic Association National Registry, lower-extremity surgeries such as THA and TKA constitute 50.7% of all orthopedic surgeries, making them the most common procedures [6]. Additionally, among patients who underwent lower-extremity surgery, older adults constituted 51.4%, compared to 44.9% among orthopedic surgery patients. Specifically, THA had the highest proportion of older patients at 73.2%, making it the most prevalent orthopedic procedure among this population. Given these demographics, it is essential to identify and mitigate risk factors associated with POD in patients following lower-extremity surgery.

Although the pathophysiological mechanisms of delirium are not completely understood, it is widely known that delirium is associated with various factors such as age, sex, and postoperative conditions [7]. Preventive interventions have been reported to reduce the occurrence of delirium drastically [8,9]. Delirium is associated with a longer length of hospital stay, higher mortality [10], decreased activities of daily living, and decreased ability to walk [11]. In addition, patients with delirium incur medical costs that are 2.5 times higher than those without delirium [12]. Delirium can significantly impact both patient outcomes and healthcare systems. Hence, it is crucial to identify risk factors and devise preventive strategies to mitigate their occurrence.

Previous studies on risk factors for delirium have involved randomized controlled trials and meta-analyses. However, existing studies have predominantly focused on specific aspects of surgical procedures and treatments, overlooking the need for comprehensive investigations encompassing patients undergoing lower-extremity surgeries such as THA and TKA. Therefore, this study aimed to identify risk factors for delirium in patients who underwent lower-extremity surgery through a systematic review and meta-analysis.

## Materials and methods

In this study, we evaluated risk factors for delirium in patients undergoing lower-extremity orthopedic surgery by integrating results from previous studies. This meta-analysis was not registered but was performed following the preferred reporting items for systematic reviews and meta-analysis (PRISMA) 2020 statement (S1 Table).

### Search strategy

To minimize bias, we conducted a literature search using two databases: MEDLINE and the cumulative index to nursing and allied health literature (CINAHL), which are widely used in the medical field [13]. The databases were last accessed on September 30, 2024.

The search terms were formulated using medical subject headings and other controlled vocabulary. They included the following therms; [deliri * OR confus * OR “transient mental disorder” OR dementia OR “cognitive disorders” OR POCD] AND [hip OR knee OR femoral OR femur OR “cruciate ligament” OR replacement OR patellar OR meniscus] AND [surg * OR operation OR operate OR operative] AND [risk OR predictor OR factor]. The first and last authors independently searched the literature using predetermined keywords. Subsequently, they independently assessed eligibility and selected articles based on predefined criteria. In cases of disagreement on a study’s eligibility, the two authors resolved the issue through discussion. Related studies were included based on the following criteria: 1) participants were patients who had undergone lower-extremity surgery, such as THA and TLA et al; 2) studies were cohort, cross-sectional, or case-control; 3) a validated delirium diagnostic/assessment tool was used to reach a diagnosis of delirium; 4) studies reported adequate data for pooling in the analysis (e.g. mean and standard deviation); 5) studies were written in English; and 6) the primary objective of the research was to evaluate risk factors for incident delirium. Exclusion criteria were 1) studies that did report data on preoperative, intraoperative, and postoperative variables, such as comorbidities, laboratory tests, and surgical procedures; 2) manuscripts for which the full text was inaccessible; 3) studies that did not include a comparison between delirium and non-delirium groups; 4) intervention studies; and 5) review articles, letters, comments, and studies assessing subsyndromal delirium, preoperative delirium, or children. The database search period was set from 1975 to July 2024.

### Quality assessment

Two independent reviewers assessed the nonrandomized studies included in this meta-analysis using the Newcastle-Ottawa Scale (NOS) [14]. This validated and recommended tool assessed the methodological quality of nonrandomized studies. NOS scores comprise of selection criteria, comparability, and outcomes. A maximum score of 9 stars indicates the highest quality, with most studies scoring between 5 and 9 stars.

### Data extraction

After the eligible studies were included, the following data were extracted: title, name of the first author, year of publication, study design, sample size, population, and average age. Next, we extracted the variables used in the analysis. Variables with significant differences were extracted

from each. Additionally, data on delirium available to nurses in clinical practice were used as variables in the analysis. For our meta-analysis, we gathered data across various categories: 10 demographic variables (e.g., age, sex), 15 comorbidities (e.g., dementia, hypertension), 18 preoperative factors (e.g., serum albumin level mini-mental state examination [MMSE] score), 21 intraoperative factors (e.g., surgical method, surgery duration), and three postoperative factors (e.g., deep vein thrombosis, pneumonia). Detailed information can be found in Table 1.

**Table 1:** The extracted variable from studies included meta-analysis.

Characteristics of patients
Age, BMI, Male, Obesity, Underweight, Polypharmacy, Smoking, Drugs, Diseases, Charlson Comorbidity Index
**Comorbidity**
Preadmission cognitive impairment, Dementia, Hypertension, Diabetes, Hyperlipidemia, Cerebrovascular accident, TIA, Parkinson’s, COPD, Cardiovascular disease, Myocardial infarction, Malignancy in < 20 years, Previous hip fracture surgery, History of delirium, Coronary heart disease
**Preoperative factors**
MMSE score, Platelet count, WBC, Lymphocyte count, CRP, NLR value, Hemoglobin, Serum albumin level, Creatinine Clearance, Blood sugar, Urea nitrogen, T3, T4, TSH, FT3, PaO^2^, PaCO^2^, HCT
**Intraoperative factors**
Hemiarthroplasty, DHS, Internal fixation, Total hip arthroplasty, Right, Left, Femoral neck, Fracture pattern; Intracapsular, Fracture pattern; Extracapsular, Length of hospital stay (Day), Time to surgery, Admission to operation duration, Duration of the procedure, Anesthesia time, Blood loss, Blood transfusion volume, General Anesthesia, Spinal Anesthesia, Subtrochanteric, Intertrochanteric, Blood transfusion
**Postoperative factors**
Complications, DVT, Pneumonia

Abbreviations

BMI, body mass index; CRP, C-reactive protein; COPD, chronic obstructive pulmonary disease; DHS, dynamic hip screw; DVT, deep vein thrombosis; FT3, free triiodothyronine; HCT, haematocrit; MMSE, mini-mental state examination; NLR, neutrophil-to-lymphocyte rate; PaCO2, partial pressure of carbon dioxide; PaO2, partial pressure of oxygen; T3, total triiodothyronime; T4, total thyroxine; TIA, transient ischaemic attack; TSH, thyroid-stimulating hormone; WBC, white blood cell

### Statistical analysis

Odds ratios (OR) or standardized mean differences (SMD) with 95% confidence intervals (CI) were calculated and pooled, and risk factors were examined in two or more included studies to estimate the association with POD. Between-study heterogeneity was tested using *I*^*2*^, as guided by the cochrane handbook for systematic reviews of interventions. A random-effects model was used to calculate pooled OR or SMD when significant heterogeneity was present (*I*^*2*^ >  50%). Otherwise, the fixed effects model was used. A statistically significant risk factor for POD was considered at a two-tailed p-value of <  0.05. In meta-analyses, including more than four studies, subgroup analyses were performed when SMD or OR had a p-value of <  0.05 and high heterogeneity. Subgroup analyses were performed using meta-regression to explore the source of heterogeneity considering sample size. In addition, a leave-one-out sensitivity analysis was performed to estimate the influence of each study.

Forest plots were used to summarize the outcomes of the meta-analysis. For risk factors demonstrated in at least 10 studies, potential publication bias was assessed using visual funnel plots for symmetry. All statistical analyses were conducted using StataIC 18.1 (Stata Corp LLC, Texas, USA).

## Results

**Fig 1:**
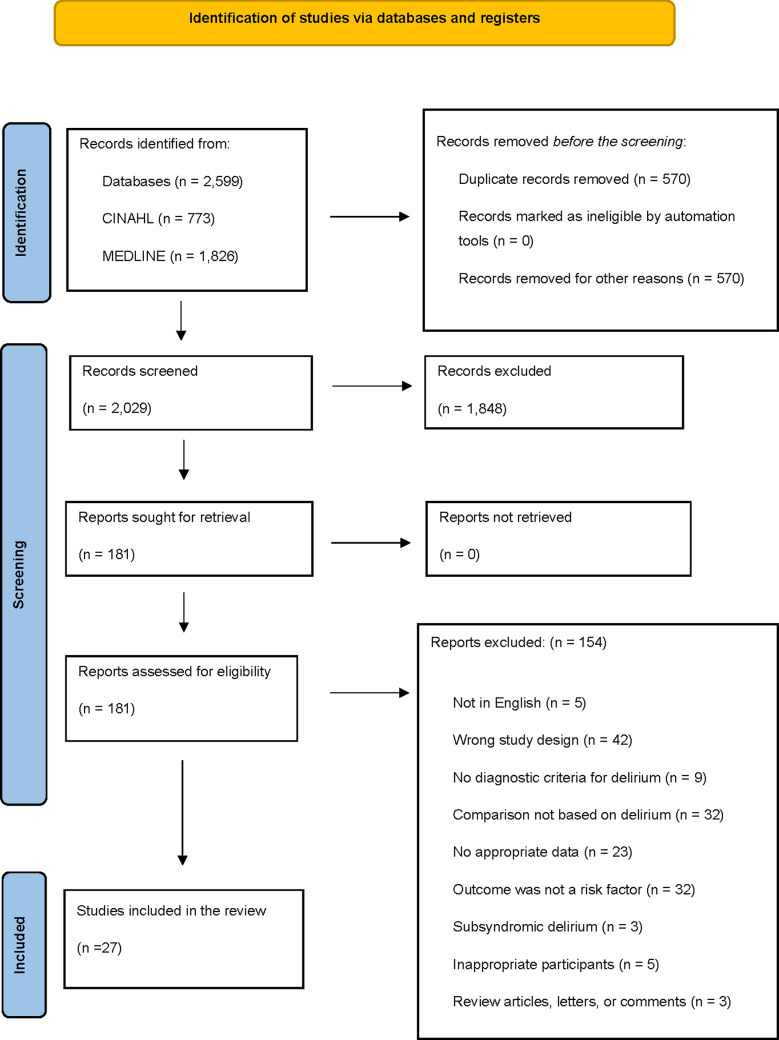
Flowchart of identification, screening, review, and selection of studies based on the PRISMA 2020 guidelines.

The database search identified 2,599 records using predefined search keywords. A total of 568 studies were excluded due to duplication and screened by reviewing the title and/or abstract following the inclusion and exclusion criteria. Consequently, 2029 studies were excluded, and the remaining 181 articles were fully reviewed for eligibility assessment. Ultimately, 27 articles met the eligibility criteria and were included in the meta-analysis (S2 Table). Fig 1 shows the process of the study search, screening, and eligibility assessment based on the PRISMA study flow diagram.

Table 2 summarizes the characteristics of this study. Regarding study design, 11 were prospective cohort studies, and 16 were retrospective cohort studies. Almost all studies used the Confusion Assessment Method to assess delirium. Furthermore, assessments were conducted, including the diagnostic and statistical manual of mental disorders, delirium observation screening scale, nursing delirium screening scale, and 4 ‘A’s Test. Studies indicated a varied incidence of POD, ranging from 6.6% to 55.9%. The mean age of patients diagnosed with delirium ranged from 67.9 ±  4.0 and 88.1 ±  5.7 years, while those without delirium ranged from 66.6 ±  5.9 to 82.6 ±  6.9 years. The quality assessment of the included studies was conducted using the NOS, which evaluates studies based on three main perspectives: selection, comparability, and outcome. Three, twelve, four, seven, and one study scored 5, 6, 7, 8, and 9 stars, respectively (Table 3).

**Table 2:** Characteristics of the studies.

Author (year)	country	Design	Assessment of Delirium	Population	Sample size	No.		Age (mean ± SD)	
						D	ND	D	ND
Li, et al. (2022) [15]	China	Retrospective cohort	CAM	patients who underwent hip replacement surgery	384	128	256	84.6 ± 5.3	71.0 ± 8.7
He, et al. (2020) [16]	China	Prospective cohort	CAM DSM-Ⅳ	patients who underwent THA for hip fracture	780	182	598	75.8 ± 8.6	73.3 ± 7.4
Bisschop, et al. (2011) [17]	the Netherlands	Prospective cohort	CAM	patients who were admitted for hip fracture	143	70	73	85.1 ± 6.7	82.6 ± 6.9
de Jong, et al. (2019) [18]	the Netherlands	Retrospective cohort	CAM DOSS DSM-IV	patients who underwent surgical treatment for proximal hip fractures	463	121	342	84 ± 8	80 ± 8
de Haan, et al. (2023) [19]	the Netherlands	Retrospective cohort	DOSS DSM-IV	Patients who underwent surgical treatment for proximal femoral fractures	1861	136	1725	85 ± 7	79 ± 11
Furlaneto, et al. (2006) [20]	Brazil	Prospective cohort	CAM	patients with hip fractures	103	30	73	82.5 ± 8.0	80.1 ± 8.5
Kong, et al. (2022) [21]	China	Retrospective cohort	CAM	patients who underwent hip fracture surgery	245	32	213	78.8 ± 7.4	71.2 ± 5.8
Venkatakrishnaish, et al. (2022) [22]	India	Prospective cohort	CAM	patients with hip fractures	110	49	61	71.6 ± 7.9	68 ± 6.9
Wang, et al. (2021) [23]	China	Retrospective cohort	CAM	patients who had been scheduled for surgery for hip fractures	272	52	220	N.A	N.A
Wang, et al. (2018) [24]	China	Retrospective cohort	CAM	patients who underwent hip surgery	306	59	247	81.9 ± 5.4	76.4 ± 8.1
Santana Santos, et al. (2005) [25]	Sweden	Prospective cohort	CAM DSM-Ⅳ	patients who underwent surgery for a fractured neck of femur	34	19	15	N.A	N.A
Nie, et al. (2011) [26]	China	Prospective cohort	CAM	patients with neck fractures of the thigh bone	123	16	107	75.0 ± 2.0	75.3 ± 0.8
Levinoff, et al. (2018) [27]	Canada	Retrospective cohort	CAM	patients who underwent surgery for a fractured hip	114	20	94	88.1 ± 5.7	82.3 ± 8.2
Kijima, et al. (2020) [28]	Japan	Retrospective cohort	CAM DSM-Ⅴ	patients who underwent unilateral or bilateral TKA	170	11	159	79.5 ± 6.9	73.0 ± 9.0
Xing, et al. (2020) [29]	China	Prospective cohort	CAM	patients who underwent hip fracture surgery	163	57	106	74.2 ± 5.2	71.8 ± 6.4
Chen, et al. (2017) [30]	China	Prospective cohort	CAM DSM-Ⅳ	patients who underwent hip or knee arthroplasty	212	35	177	81.8 ± 6.8	72.2 ± 5.1
Jeon, et al. (2021) [31]	Korea	Retrospective cohort	Nu-DESC	patients who underwent hip replacement surgery	231	104	127	83.5 ± 6.9	79.0 ± 8.5
Kim, et al. (2017) [32]	Korea	Prospective cohort	CAM	patients who were diagnosed as having femoral neck fracture or intertrochanteric hip fracture	74	37	37	81.8 ± 6.8	80.8 ± 6.7
Zhang, et al. (2022) [33]	China	Prospective cohort	CAM MADS	patient who underwent total knee replacement due to severe osteoarthritis	268	42	226	70.3 ± 5.0	66.6 ± 5.9
Rajeev, et al. (2022) [34]	UK	Prospective cohort	4AT	Patients who sustained a proximal femur fracture and underwent surgery	598	175	423	84.8 ± 16.8	81.2 ± 17.7
Chu, et al. (2021) [35]	China	Retrospective cohort	CAM	patients who underwent surgery for hip fracture	462	74	388	67.9 ± 4.0	67.4 ± 2.6
Xu, et al. (2021) [36]	China	Retrospective cohort	CAM	patients who underwent surgical treatment for hip fracture	568	82	486	79.5 ± 5.3	70.2 ± 5.1
Liu, et al. (2023) [37]	USA	Retrospective study	Nu-DESC	patients who underwent hip surgery	97	32	65	79.8 ± 9.0	75.2 ± 8.3
Song, et al. (2024) [38]	China	Retrospective study	CAM	patients who underwent TKA	446	79	367	75.6 ± 4.3	68.8 ± 5.3
Wang, et al. (2023) [39]	China	Retrospective study	CAM	patients who underwent THA	151	26	125	78.6 ± 7.3	63.3 ± 2.7
Riemenschneider, et al. (2024) [40]	Germany	Retrospective study	ICDSC	patients who underwent surgical treatment for femoral neck fracture	412	75	337	84.8 ± 8.6	80.6 ± 9.6
Hu, et al. (2023) [41]	Brazil	Retrospective study	CAM	patients who underwent THA	254	49	205	71.5 ± 6.9	67.3 ± 5.0

Abbreviations CAM, The Confusion Assessment Method; D, delirium; DOSS, Delirium Observation Screening Scale; DSM, Diagnostic and Statistical Manual of Mental Disorders; ICDSC, Intensive Care Delirium Screening Checklist; ND, non-delirium; Nu-DESC, Nursing Delirium Screening Scale; MADS, Memorial Delirium Assessment Scale; TKA, Total Knee Arthoplasty; THA, Total Hip Arthroplasty; 4AT, 4 ‘A’s Test

**Table 3:** Newcastle Ottawa Scale (NOS) scoring for the cohort studies.

	Selection				Comparability		Outcome			
Author	Representativeness of the exposed cohort	Selection of the non exposed cohort	Ascertainment of exposure	Demonstration that outcome of interest was not present at start of study	Comparability of cohorts on the basis of the design or analysis		Assessment of outcome	Was follow up long enough for outcomes to occur	Adequacy of follow up of cohorts	Total
Li, et al. (2022)	1	1	1	0	0	0	1	0	1	5
He, et al. (2020)	1	1	1	1	1	0	1	1	1	8
Bisschop, et al. (2011)	1	1	1	0	1	0	1	0	1	6
de Jong, et al. (2019)	1	1	1	0	1	0	1	1	1	7
de Haan, et al. (2023)	1	1	1	0	1	0	1	0	1	6
Furlaneto, et al. (2006)	1	1	1	0	0	0	1	1	1	6
Kong, et al. (2022)	1	1	1	1	0	0	1	0	1	6
Venkatakrishnaiah, et al. (2022)	1	1	1	0	1	0	1	0	1	6
Wang, et al. (2021)	1	1	1	1	1	0	1	1	1	8
Wang, et al. (2018)	1	1	1	1	0	0	1	0	1	6
Santana Santos, et al. (2005)	1	1	1	1	1	0	1	1	1	8
Nie, et al. (2011)	1	1	1	1	0	0	1	1	0	6
Levinoff, et al. (2018)	0	1	1	0	1	0	0	1	1	5
Kijima, et al. (2020)	1	1	1	0	1	0	1	0	1	6
Xing, et al. (2020)	1	1	1	1	1	0	1	1	1	8
Chen, et al. (2017)	1	1	1	1	1	0	1	0	1	7
Jeon, et al. (2021)	1	1	1	0	1	0	1	0	1	6
Kim, et al. (2017)	1	1	1	1	1	1	1	1	1	9
Zhang, et al. (2022)	1	1	1	0	1	1	1	0	1	7
Rajeev, et al. (2022)	1	1	1	0	0	0	1	0	1	5
Chu, et al. (2021)	1	1	1	1	0	0	1	0	1	6
Xu, et al. (2021)	1	1	1	1	1	0	1	1	1	8
Liu, et al. (2023)	1	1	1	0	1	0	1	1	1	8
Song, et al. (2024)	1	1	1	0	1	0	1	1	1	8
Wang, et al. (2023)	1	1	1	0	0	0	1	1	1	6
Riemenschneider, et al. (2024)	1	1	1	0	0	0	1	1	1	6
Hu, et al. (2023)	1	1	1	0	1	0	1	1	1	7

### Meta-analysis

In the 27 studies included in the analysis, data from 9,044 participants were synthesized, of whom 1,792 had delirium, and 7,252 did not. The sample size of each study ranged from 34 to 1,861 participants. The extracted 67 variables were subjected to a meta-analysis (S3 Table). The results of the random-effects Mantel-Haenszel model analysis are shown in S4 Table. Consequently, this meta-analysis revealed 20 risk factors for patients with POD undergoing orthopedic surgery (Table 4).

**Table 4:** Risk factors for POD identified by meta-analysis.

variable	Studies included	participants included	SMD	OR	95%CI	p value	*I2*
**Characteristics of patients**							
Age	25	8928	0.8		0.56–1.03	0	94.4
Polypharmacy	2	2514		1.47	1.19–1.82	0	0
**Cormobidity**							
Preadmission cognitive impairment	4	793		3.76	1.57–9.02	0.003	57
Dementia	4	2760		4	2.09–7.67	0	72.4
TIA	2	2514		1.69	1.22–2.32	0.001	0
Parkinson	2	2514		2.32	1.56–3.44	0	44.8
Cardiovascular disease	4	3524		1.4	1.16–1.68	0.001	0
Myocardial infarction	2	2514		1.4	1.01–1.95	0.047	0
History of delirium	3	1275		12.64	8.75–18.27	0	40.5
**Preoperative factors**							
MMSE score	4	1197	-0.59		-1.05–-0.13	0.012	87.8
CRP	5	1680	0.47		0.05–0.88	0.028	89.3
Serum albumin level	6	1653	-0.69		-1.09–-0.29	0.001	89
TSH	2	813	-1.83		-2.66–-1.01	0	91.3
FT3	2	813	-0.37		-0.62–-0.12	0.003	29.5
**Intraoperative factors**							
Length of hospital stay (Day)	7	1452	0.47		0.18–0.76	0.001	82
duration of the procedure	14	3812	0.53		0.16–0.90	0.005	95
Anaesthesia time	7	1702	0.16		0.05–0.28	0.005	0
General Anaesthesia	8	2713		1.28	1.04–1.58	0.021	0
Spinal Anaesthesia	9	2856		0.79	0.65–0.97	0.023	0
Blood transfusion	4	1046		2.03	1.02–4.08	0.045	69.9

Abbreviations

CRP, C-reactive protein; FT3, free triiodothyronine; MMSE, mini-mental state examination; TIA, transient ischemic attack; TSH, thyroid

### Characteristics of the patients

**Fig 2:**
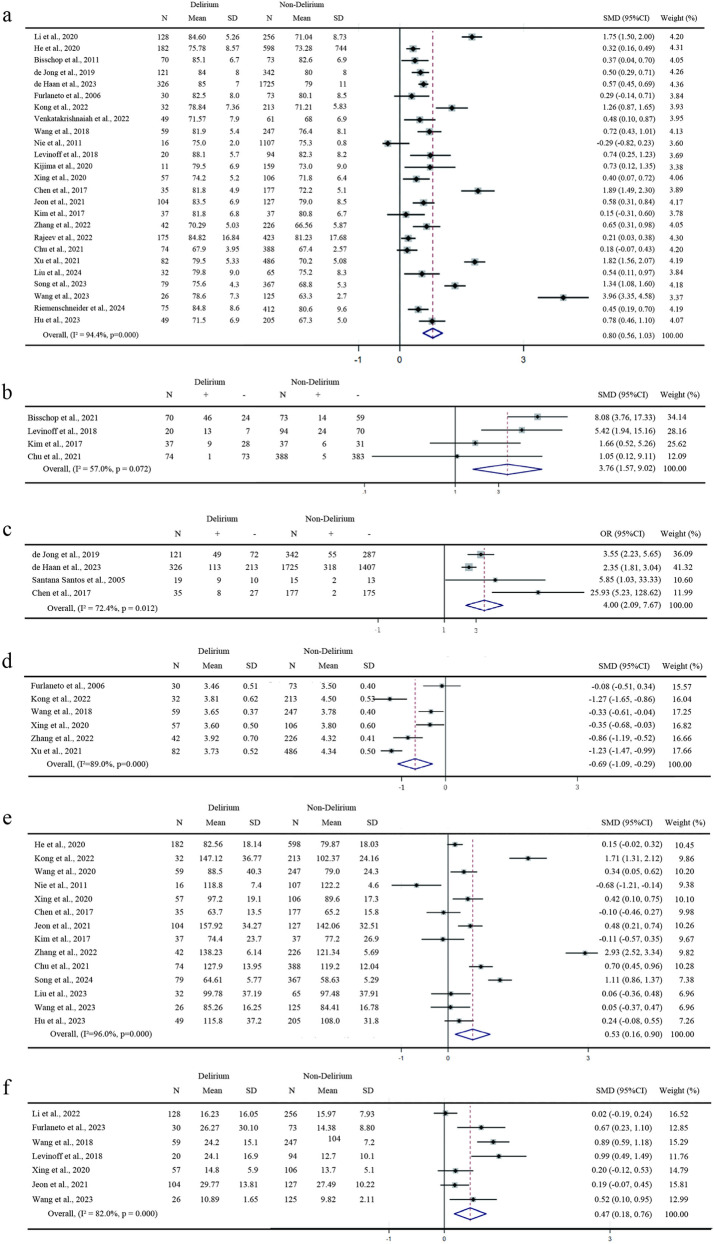
Forest plot for risk factors of postoperative delirium. The forest plot indicates the sample size, effect size, 95% confidence interval (95% CI), and weighted effect size of each study. This includes boxes, sample size, filled circle, effect size, horizontal lines, the apex of the rhombus, weighted effect size, rhombus width, and 95% CI. a. Age. b. Preoperative cognitive impairment. c. Dementia comorbidity. d. Preoperative serum albumin level. e. Length of hospital stay. f. Duration of the procedure.

Ten variables based on patient characteristics were included in this meta-analysis. Age was identified as a risk factor for POD in 25 studies. Consequently, patients with delirium were older than those without delirium, reaching statistical significance (SMD 0.8, 95%CI 0.56–1.03, p <  0.001, *I*^*2*^ =  94.4%) (Fig 2a). Additionally, polypharmacy, as identified by two studies, was found to be independently associated with POD (OR 1.47, 95% CI 1.19–1.82, p <  0.001, *I*^*2*^ =  0). However, variables such as sex, body mass index, obesity, low weight, smoking status, number of drugs, medical history, and the Charlson Comorbidity Index showed no statistically significant association with POD.

### Comorbidity

Fifteen variables related to comorbidities were included in this meta-analysis. The comorbidity variables that were statistically significant in terms of their association with POD were as follows: cognitive impairment (OR 3.76, 95%CI 1.57–9.002, p =  0.003, *I*^*2*^ =  57.0%) (Fig 2b), cardiovascular disease (OR 1.4, 95%CI 1.16–1.68, p =  0.001, *I*^*2*^ =  0), transient ischemic attack (OR 1.69, 95%CI 1.22–2.32, p =  0.001, *I*^*2*^ =  0), myocardial infarction (OR 1.40, 95%CI 1.01–1.95, p =  0.044, *I*^*2*^ =  0), Parkinson disease (OR 2.15, 95%CI 1.13–4.08, p =  0.02, *I*^*2*^ =  44.5%), dementia (OR 4.00, 95%CI 2.09–7.67, p <  0.01, *I*^*2*^ =  72.4%) (Fig 2c), history of delirium (OR 12.88, 95%CI 7.85–21.13, p <  0.01, *I*^*2*^ =  40.5%). Hypertension, diabetes mellitus, hyperlipidemia, cerebrovascular disease, coronary disease, chronic obstructive pulmonary disease, malignancy, and surgical experience with femoral neck fractures were not statistically significant in terms of their association with POD.

### Preoperative factors

Nineteen preoperative factors were included in this meta-analysis. Preoperative factors that showed statistically significant differences were MMSE score (SMD -0.59;95%CI -1.05– -0.13;p =  0.012; I² =  87.8%), thyroid-stimulating hormone (TSH) (SMD -0.37; 95%CI -2.66– -1.01; p <  0.001; *I*^*2*^ =  91.3%), C-reactive protein (CRP) (SMD 0.47; 95%CI 0.05–0.88; p <  0.028; *I*^*2*^ =  89.3%), and serum albumin level (SMD -0.69;95%CI -1.09– -0.29;p =  0.001; I² =  89.0%) (Fig 2d). In addition, free triiodothyronine (FT3) was associated with POD (OR -0.37;95%CI -0.62– -0.12;p =  0.003; *I*² =  29.5%), with this difference being statistically significant. Preoperative factors that did not exhibit statistically significant differences or associations with POD included platelet count, white blood cell count, lymphocyte count, c-reactive protein, neutrophil-to-lymphocyte ratio, hemoglobin, hematocrit, blood sugar, blood urea nitrogen, creatinine, triiodothyronine, thyroxine, partial pressure of oxygen, and partial pressure of carbon dioxide.

### Intraoperative and postoperative factors

In our analysis of 21 intraoperative variables, statistically significant differences were observed in the length of hospital stay, anesthesia time, and duration of the procedure (SMD 0.47; 95% CI 0.18–0.76; p =  0.001; *I*² =  82.0%, SMD 0.15; 95% CI 0.02–0.27; p =  0.026; *I*² =  0.0%, SMD 0.53; 95% CI 0.16–0.90; p =  0.005; *I*² =  95.0%) (Figs 2e and 2f). Additionally, comorbidity variables that were statistically significant in terms of their association with POD were as follows: general anesthesia and POD (OR 1.28; 95% CI 1.04–1.59; p =  0.02; *I*² =  0), local anesthesia (OR 0.79; 95% CI 0.65–0.97; p =  0.022; *I*² =  0) and blood transfusion (OR 2.03; 95% CI 1.02–4.08; p =  0.045; *I*² =  69.9). No statistically significant differences were found in the three postoperative variables included in the meta-analysis between patients with and without delirium.

### Publication bias and subgroup analyses

**Fig 3:**
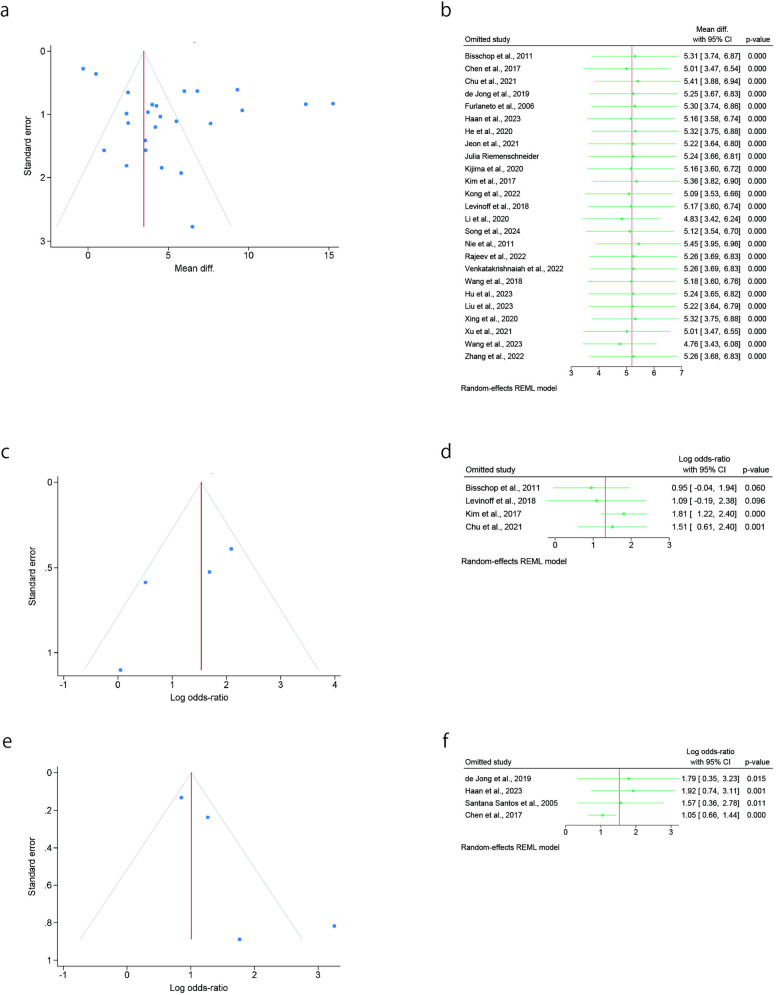
Funnel plots with the log odds ratio or MD and leave-one-out sensitivity analyses for characteristics of patients and comorbidity variables. a. Funnel plot for age. b. Sensitivity analysis for age. c. Funnel plot for preoperative cognitive impairment. d. Sensitivity analysis for preoperative cognitive impairment. e. Funnel plot for dementia comorbidity. d. Sensitivity analysis for comorbidity of dementia.

**Fig 4:**
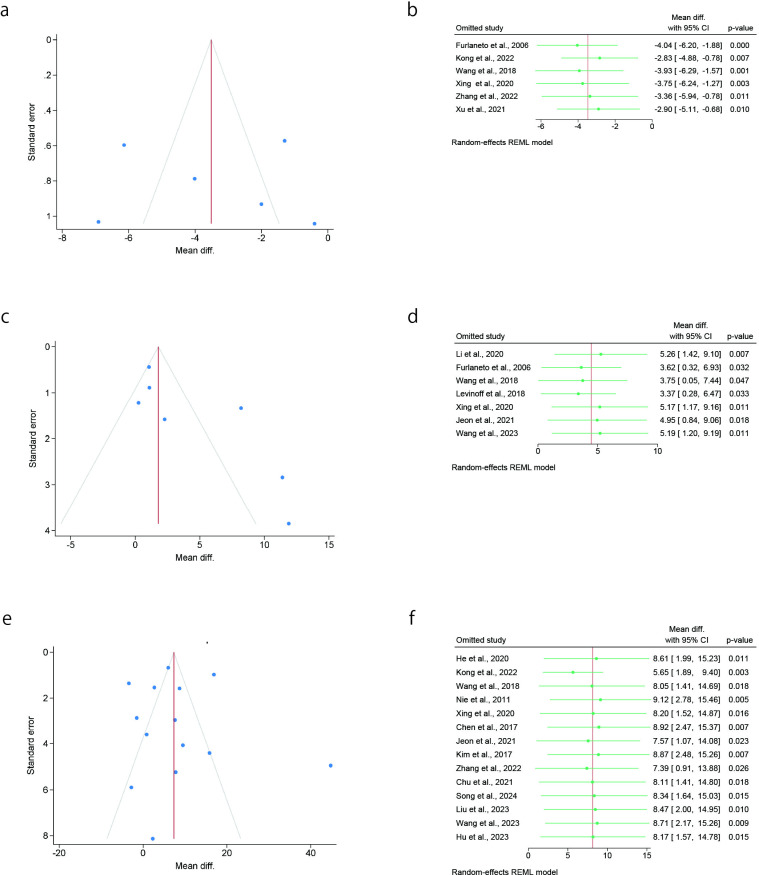
Funnel plots with the log odds ratio or MD and leave-one-out sensitivity analyses for pre and intraoperative variables. a. Funnel plot for preoperative serum albumin level. b. Sensitivity analysis for preoperative serum albumin level. c. Funnel plot for the length of hospital stay. d. Sensitivity analysis for the length of hospital stay. e. Funnel plot for the duration of the procedure.

We examined the funnel plot of variables analyzed in more than four studies and observed high heterogeneity. These variables were also subjected to subgroup analyses, including meta-regression analysis and leave-one-out sensitivity analyses. The variables included age, dementia, preadmission cognitive impairment, preoperative albumin, duration of the procedure, and length of hospital stay (days). We visually inspected the funnel plot and found that these six variables were asymmetrical, indicating a publication bias. Table 5 shows the results of the meta-regression analysis performed by considering sample size as the covariate. No effect size not statistically significantly associated with sample size for all variables. In the leave-one-out sensitivity analysis, the SMD or OR omitting each study individually did not fluctuate, and one study did not have a large influence the variables (Figs 3 and 4).

**Table 5:** The results of meta-regression analysis performed by considering sample size as covariance.

Variables	Coefficient	Standard error	p-value	95% Confidence Interval
Age	0.0005484	0.0019772	0.782	-0.0033269–0.0044236
Dementia	-0.000637	0.000511	0.213	-0.0016386–0.0003646
Preadmission cognitive impairment	-0.002863	0.0040449	0.479	-0.0107909–0.0050648
Preoperative albumin	-0.0098812	0.00637	0.121	-0.0223663–0.0026038
Duration of the procedure	0.0033516	0.0179055	0.852	-0.0317426–0.0384459
Length of hospital stay (Day)	-0.0177389	0.0194115	0.361	-0.0557846–0.0203069

## Discussion

This study revealed the risk factors for delirium in patients who underwent lower-extremity surgery through a systematic review and meta-analysis. We identified 27 studies, including 11 prospective and 16 retrospective cohort studies, and extracted 67 factors associated with POD from characteristics, comorbidities, and preoperative, intraoperative, and postoperative factors. Of the nine numeric factors, statistically significant differences were observed between patients with and without delirium. Additionally, 11 of the 37 categorical factors were significantly associated with POD. A systematic review and meta-analysis of patients who underwent knee and hip replacement reported that 29 preoperative and intraoperative variables, including medical comorbidities and laboratory tests, were evaluated, with 18 factors identified as risk factors for POD. According to the meta-analysis study by Rong et al., 16 studies focusing on patients who underwent knee arthroplasty were included [42]. Conversely, although the participants in this study underwent lower-extremity surgery, four of the 27 studies included studies targeted patients with knee joint disease. We included observational studies that comprehensively examined preoperative to postoperative factors in the meta-analysis. As a result, most of the 16 studies included in the previous meta-analysis have been excluded during the first or second screening of this study.

In previous meta-analysis studies on orthopedic procedure, age, cognitive impairment, cerebrovascular disease, laboratory values such as total albumin, and surgery-related variables were identified as risk factors for POD, which is consistent with our study [42,43]. However, some of these risk factors demonstrated high heterogeneity. Similarly, in this study, high heterogeneity was observed in age, cognitive impairment, dementia, MMSE score, albumin and TSH levels, length of hospital stay, and duration of the procedure. Therefore, this study used meta-regression analysis to explore the sources of heterogeneity, considering sample size. In addition, a leave-one-out sensitivity analysis was performed to estimate the influence of each study. The sample size significantly influenced the findings, and the analysis demonstrated robustness. The exclusion and subsequent reanalysis of each study did not lead to fluctuating results.

### Characteristics of patients

The age of patients with delirium who underwent surgery was higher than those without delirium. Delirium may result from increased comorbidity, organic brain changes, and declining kidney/metabolic functions in older patients. Furthermore, age-related alterations in central acetylcholine and neurotransmitter levels, particularly dopaminergic excess, may contribute to POD [44]. Changes in neurotransmitter levels such as acetylcholine, norepinephrine, epinephrine, and gamma-aminobutyric acid with age are associated with POD occurrence [45,46]. Consequently, older age is a significant risk factor for POD in individuals undergoing lower-extremity surgery.

Regarding polypharmacy, Catic et al. reported a 39% risk of POD in geriatric patients, indicating a high rate [47]. Polypharmacy can cause side effects and unexpected changes by altering the pharmacokinetics and pharmacodynamics of comorbidities. Moreover, the interplay between medications and underlying diseases, has been linked to cognitive impairment in geriatric patients [48]. Specifically, acetylcholine function can be inhibited by the anticholinergic effects of certain medications, including antiarrhythmics, antipsychotics, sleep aids, and analgesics. This inhibition creates an imbalance, as acetylcholine normally has an antagonistic effect on dopamine. Excessive dopamine secretion due to this imbalance can lead to hallucinations and psychomotor disturbances, which are symptomatic of delirium [49].

### Comorbidity

This study found an association between prior cognitive impairment and dementia and POD. Pathophysiologically, the neurological degeneration process of delirium shares similarities with dementia, characterized by an excessive inflammatory response and cholinergic dysfunction [50]. In addition, patients with dementia experiencing psychobehavioural symptoms may undergo sleep disturbances and electrolyte imbalances, indirectly heightening their susceptibility to delirium [51]. Previous studies have revealed that delirium has a clear and complex association with dementia [52] and that delirium severity is significantly associated with cognitive impairment [53]. In this study, the OR for the association between delirium and previous cognitive impairment and dementia were notably high, at 3.76 and 4.00, respectively.

Delirium is a common complication of Parkinson’s disease that increases the rate of delirium occurrence during hospital admission [54]. Parkinson’s disease-related neuronal decline leads to dysregulated neurotransmitter secretion, including dopamine and acetylcholine, which can precipitate delirium [55]. Consequently, Parkinson’s disease is linked to an increased susceptibility to delirium.

This study found that the risk factors associated with delirium were myocardial infarction, cardiovascular disease, and transient ischemic attack, all of which affect cerebral perfusion. Delirium is caused by brain hypoperfusion, which reduces the synthesis of acetyl coenzyme A, glutamate, and acetylcholine during the citric acid cycle. Reduced cholinergic and glutaminergic activity in the brain may be responsible for delirium [56,57]. As a result, delirium is more likely to occur when brain perfusion is a temporarily reduced by conditions such as myocardial infarction and cardiovascular disease, which hinder effective circulation throughout the body. Transient ischemic attack also contributes to the increased likelihood of delirium due to its impact on cerebral blood flow [56,58,59]. Furthermore, the OR for a history of delirium, estimating its association in this study, was notably high value at 12.88. This underscores the importance of heightened vigilance for delirium in patients with a history of the condition.

### Preoperative factors

Preoperative factors represent the general condition of patients and are associated with various postoperative complications. The SMD of MMSE scores between patients with and without delirium was statistically significant, at -0.34. Additionally, preoperative albumin levels are an important factor in postoperative complications, as lower levels have been associated with POD [60]. Poor nutritional status, as evidenced by reduced albumin, can lead to muscle weakness, decreased immune function, and an increased risk of infection [61], all of which may precipitate POD. Additionally, Espaulella et al. reported that postoperative complications, including delirium, were reduced by consuming nutritional supplementary food [62]. Accordingly, preoperative albumin levels are likely associated with POD.

Regarding TSH and FT3 levels, which indicate thyroid function, patients with latent hyperthyroidism (TSH <  0.45 mU/L) are at high risk of cognitive function impairment within 5 years of onset [63]. Orthopedic surgical procedures take a long time to increase the stress response in the postoperative human body. This response disrupts the hypothalamic-pituitary-thyroid axis and affects thyroid function [64]. Thyroid hormone is essential for maintainsing normal function of the central nervous system and is closely associated with psychological activity [63]. Consequently, thyroid hormone levels in the central nervous system affect patients and increase the risk of POD. Therefore, thyroid function, including TSH and FT3 levels, is considered a risk factor for POD.

### Intraoperative factors

A prolonged surgical duration may increase operative stress and inflammatory cytokine levels [65]. Elevated levels of inflammatory cytokines are associated with POD [66]. Extended anesthesia, through prolonged surgical duration, exposes the body to stress induced by surgical trauma for an extended period [67]. This stress can lead to metabolic dysfunction, which increases the possibility of norepinephrine and acetylcholine involvement in central nervous system dysfunction. Consequently, an imbalance of norepinephrine and acetylcholine excites the brain, causing POD [68,69]. Pathophysiologically, POD is hypothesized to result from impaired brain autoregulation. Prolonged surgical duration exacerbates hypercapnia, hypothermia, and increased blood loss, diminishing autoregulation and the development of POD [42]. General anesthesia, including propofol, influences neurocyte processes and is frequently associated with POD [70]. In a previous study comparing general and spinal anesthesia, the general anesthesia group showed prominent psychological changes, but the spinal anesthesia group did not. General anesthesia may lead to hyperventilation, diminished cardiac output, cerebral blood flow, hypoxia, and cerebrovascular contraction after surgery, all contributing to the development of POD [71]. In contrast, the OR for the association between delirium and spinal anesthesia was statistically significant, with an OR of below 0.76 in this study. Spinal anesthesia is prohibited when patients undergo extensive surgical procedures or when a risk of bleeding is observed. Patients undergoing spinal anesthesia surgery may experience less invasiveness than those under general anesthesia. Consequently, major invasive surgery, typically involving the secretion of more inflammatory cytokines, emerges as a risk factor for POD [72]. Therefore, the decreased OR for POD with spinal anesthesia may not indicate a reduced risk of POD.

We identified 20 factors associated with POD after orthopedic surgery. The insights from this study can contribute to the prediction and prevention of POD following orthopedic surgery. Clinical guidelines recommend identifying patients at high risk of POD and implementing early preventive care [73,74]. Most risk factors identified in this study were data that medical professionals could extract from medical records during preoperative and intraoperative periods. Therefore, medical professionals can identify patients at high risk of POD early by collecting, sharing, and assessing information about these risk factors. Interprofessional collaborative interventions are essential to prevent POD [75]. This study suggests that POD is associated with nutritional conditions, such as preoperative serum albumin levels, and hospital stays length. Speech therapists assess swallowing function, while physical therapists and nurses adjust eating positions to improve nutritional conditions. Additionally, maintaining circadian rhythm through early mobilization, cognitive stimulation, and sleep promotion is important for shortening the length of hospital stay. Therefore, POD preventive care should be provided as a systematic interdisciplinary care program through cooperation among physicians, nurses, pharmacists, and physiotherapists.

This study had some limitations. First, this meta-analysis included diverse orthopedic surgeries, study designs, sample sizes, and patient characteristics. Consequently, this study had substantial heterogeneity. Second, the participants of this study were patients who underwent lower-extremity surgery; in 23 of the 27 studies, the participants were patients with hip joint disease. Therefore, it is possible that not all lower-extremity surgery patients were included in this study. Third, 13 of the extracted studies were conducted in one country; therefore, the synthesized population is likely to be biased. Finally, the number of postoperative factors was low. Postoperative parameters, including vital signs and blood gas data, need to be considered because postoperative hemodynamic status and electrolyte changes are associated with POD.

## Conclusion

In conclusion, 20 factors were revealed to be associated with POD following orthopedic surgery. Several factors identified in this study were related to abnormal neurotransmitter secretion and reduced cerebral perfusion, which are associated with POD. Furthermore, preventive measures against POD risk factors may reduce its occurrence. Consequently, medical professionals must gather preoperative and postoperative information to identify patients who are at a high risk of POD. Therefore, this study’s results may aid in predicting and preventing POD after orthopedic surgery.

## Supporting information

S1 Table The Preferred Reporting Items for Systematic Reviews and Meta-Analysis (PRISMA) 2020 statement checklist. (DOCX)

S2 Table List of studies identified in the literature search. (XLSX)

S3 Table Extracted risk factors for POD from studies included in meta-analysis. (XLSX)

S4 Table Result of meta-analysis for extracted risk factors for POD from studies included in meta-analysis. (XLSX)
